# Multidisciplinary management of immunotherapy‐related adverse events in solid tumors: An inter‐institutional and telemedicine‐based working team

**DOI:** 10.1002/cam4.7403

**Published:** 2024-07-05

**Authors:** Alessandro Iaculli, Michele Ghidini, Francesco Locati, Laura Chiappa, Giuseppe Nastasi, Gianpiero Fasola, Francesco Grossi, Ornella Garrone, Valeria D. Tozzi

**Affiliations:** ^1^ Oncology Unit ASST Bergamo Est Seriate Bergamo Italy; ^2^ Oncology Unit Fondazione IRCCS Ca’ Granda Ospedale Maggiore Policlinico Milan Italy; ^3^ ASST Bergamo Est Seriate Bergamo Italy; ^4^ Fondazione IRCCS Ca’ Granda Ospedale Maggiore Policlinico Milan Italy; ^5^ Oncology Department Azienda Sanitaria Universitaria Friuli Centrale (ASUFC) Udine Italy; ^6^ Oncology Unit Università dell'Insubria, ASST dei Sette Laghi Varese Italy; ^7^ Cergas SDA Bocconi School of Management Milan Italy

**Keywords:** immune‐related adverse events, immunotherapy, key performance indicators, multidisciplinary team, teleconsultation

## Abstract

**Background:**

Although immune checkpoint inhibitors (ICIs) show a more favorable toxicity profile than classical cytotoxic drugs, their mechanism of action is responsible for peculiar new toxicities. There is an urgent need for a multidisciplinary approach to advice on how to manage organ‐specific toxicities.

**Methods:**

Our project aims to integrate the practices of two different hospitals into a single Italian regional collaborative model to treat immune‐related adverse events (irAEs). The team structure is a multi‐professional and multidisciplinary cooperative network that consists of different medical specialists. The team referrer is the medical oncologist and an existing telematic platform is used for specialists' cooperation. The leading oncologist first evaluates patients' clinical condition, therefore team intervention and teleconsultation are planned to activate proper management. After a first phase structured for general setting, outcomes analysis, data collection, and identification of critical issues, it is planned to define appropriate key performance indicators (KPIs) in quality, structure, process, and outcome settings. Therefore, a second phase would serve to implement KPIs. In the third phase, the proposal for the enlargement of the network with the extension to more centers in the context of the Regional Health Service will be performed.

**Discussion:**

The multidisciplinary management of irAEs based on telemedicine fits into the debate on the renewal of healthcare systems and the push for change toward multidisciplinary with the rising use of telemedicine. To our knowledge, this is the first project reporting a multi‐institutional experience for change of service in irAEs management.

## 1 INTRODUCTION

The development of immune checkpoint inhibitors (ICIs) and the introduction of immunotherapy to treat solid tumors are the main advancements in oncology in the last decade. Acting on regulatory inhibitors of the adaptive immune system they counteract immune suppressive tumor microenvironments, thus enabling T cells to develop an effective anticancer response.^1^ Although these drugs generally show a more favorable toxicity profile as compared to classical cytotoxic drugs and tyrosine kinase inhibitors, their unique mechanism of action is both responsible for atypical response patterns and peculiar new toxicity features. These new adverse events (immune‐related adverse events, irAEs) are challenging for the oncologists as compared to those they were used to manage. Mechanisms responsible for the development of inflammation—driven by CD8 T cells—are the same involved in the therapeutic effects; however, the exact pathogenesis is unknown.^1^ Currently, there are no validated predictive factors and the identification of reliable predictors is an emerging area of investigation. Several clinical markers have been investigated: proposed markers are high body mass index (BMI), some human leukocyte genotypes (HLA), baseline modifications from normal ranges in levels of interleukin‐6 and 17 (IL‐6 and IL‐17), prior use of NSAIDs, and gut microbiome for diarrhea and colitis.^1^

Most cases of irAEs arise in the first 4 months, but they can have heterogeneous and hardly predictable time‐scale and modality of onset, with early appearance (<2 months, generally cutaneous and gastrointestinal irAEs) or late (>2 months in case of pulmonary, endocrine, and renal) as compared to classic toxicities which usually present in a clear dose/time correlation.^2^

Occasionally they can be extremely severe and rapidly worsening, with ~10% of patients receiving anti‐PD‐1 developing severe or fatal grade 3–4 toxicities. Globally the risk of toxic death from ICIs treatment is moderate, and treatment‐related fatalities have been reported in up to 2% of patients included in clinical trials. Meta‐analysis of 112 studies and 19,217 patients reported a mortality rate of 0.36% for anti‐PD‐1, 0.38% with anti‐PD‐L1, 1.08% for anti‐CTLA‐4, and 1.23% caused by combinations.^3^ In the WHO pharmacovigilance registry VigiLyze from 2009 to 2018, 613 cases of fatal irAEs were reported. The most common cause of death from anti‐CTLA‐4 was colitis (70%), whereas pneumonia (35%), hepatitis (22%), and neurological toxicities (15%) were the main causes of death from anti‐PD‐1/PD‐L1. For associations of anti‐CTLA‐4 and anti‐PD‐1, the causes of death were due to colitis (37%) and myocarditis (25%), the latter representing the higher mortality rate event (40%).^3^ However, as cancer immunotherapy continues to evolve and the total number of indications continues to expand, the burden of all‐grade irAEs and fatal events will continue to rise, thus increasing the number of deaths otherwise preventable with early diagnosis and proper treatment.^3^ A study estimated that in 2018 in the US 44% of patients with metastatic solid or hematological tumors were eligible for ICIs, and as of November 2020, there were 55 FDA‐approved indications for ICIs. Every organ or system can be affected; currently no clinical or biochemical markers/ factor may predict differential incidence and prevalence by organ or drug.^4^ Meta‐analysis reported an overall incidence of <75% with anti‐CTLA‐4 monotherapy, while phase 3 trials of anti‐PD‐1/PD‐L1 agents indicated an incidence of ≤30%. Skin, endocrine system, and colon are by far the most frequently affected, but pattern, incidence and severity can widely vary depending on the type of ICI (anti‐CTLA‐4 or anti‐PD‐1/PD‐L1) and schedule (monotherapy or combination). Meta‐analysis from Bertrand et al.^5^ (22 studies and 1265 patients) reported for anti‐CTLA4 an incidence of 72% for all‐grade irAEs with a 24% rate for grade ≥3 toxicities, also reporting a correlation with a dose for ipilimumab (grade 3 and grade ≥3 of 61%/17% for dose of 3 mg/kg and 79%/31% for 10 mg/kg). More frequent events were cutaneous (44%), gastrointestinal (35%), endocrine (6%), and hepatic (5%). Recent pharmacovigilance studies from the post‐marketing database focused on a higher incidence of neurological adverse events than reported in phase III studies.^6^ Anti PD‐1/PDL‐1 are generally better tolerated: in the metanalysis from Wang more common irAEs were diarrhea (9.47%), hypothyroidism (6.07%), and transaminase elevation (3.39%); grade >3 events were transaminase elevation (0.75%), pneumonia (0.67%) and diarrhea (0.59%); a trend to higher incidence of grade ≥3 irAEs seems to emerge for anti‐PD‐1 as compared to anti‐PD‐L1.^5^ Combination treatment is associated with increased risk: in Checkmate‐067 study (945 patients with advanced melanoma receiving nivolumab, ipilimumab, or combination), incidence of all‐grades and grade >3 irAEs were 87.9% and 39.6% for combo arm, as compared to 62% and 7.7%, respectively, in nivolumab arm and 73.6% and 18.6% for ipilimumab.^7^

Patients with pre‐existing dysimmune disorders often have been excluded from trials, owing to concerns that autoimmune disorders could be worsened by ICIs. A systematic review of patients with pre‐existing autoimmune conditions who received ICIs did not report an increased rate of de novo irAEs, while disease flare‐ups were shown to be more common during ICI treatment (50% of patients).^8^ Indeed, an ongoing NCTN trial is currently evaluating the use of ICIs in patients with underlying autoimmune conditions (ECTCN10204). All these unique and atypical features of immuno‐mediated toxicities put a challenge to oncologists. Early detection of clinical signs which are often not immediately recognizable requires strict patient monitoring and the skill to frame, grade, and treat them from a broad and multidisciplinary point of view, while predictive tools are lacking. Clinicians need to make a careful basal patient assessment, while management, surveillance, and treatment are rapidly becoming a new benchmark for the global modeling of patient pathways, focusing on a multispecialistic and highly coordinated vision: clinical management of such protean events is a challenge of great complexity. There is an urgent need for a multidisciplinary approach to correctly report and manage organ‐specific toxicities. Accordingly, organizational adjustments are needed in health services basing on evidence produced in clinical practice.

## 2 MODELS OF MANAGEMENT OF irAEs

Optimal management of irAEs has yet to be defined: experiences published to date are mainly position papers, single‐center datasets, consensus conferences, and guidelines. The evidence so far underlines the need for constant and strict cooperation between specialists and oncologists in a multidisciplinary management approach to ameliorate relevant clinical outcomes such as time of recovery, hospitalization rates, and treatment discontinuation rates. There are consolidated models of “vertical” integration between healthcare providers focused on the management of solid tumors in different phases or from different points of view, as Oncology has historically represented a benchmark for multidisciplinary decisional processes. Examples are Multidisciplinary Teams (MDT) and Disease Units which have been created and implemented from the first normative references made.^9^, ^10^, ^11^, ^12^, ^13^, ^14^, ^15^, ^16^ On the other hand, there are also models of “horizontal” integration, by organ or disease site: an anticipatory paradigm has historically been cardio‐oncology, which over the years has produced an abundant amount of literature and is today a consolidated field of integration, with regularly updated guidelines.^17^ In the era of next generation sequencing, new massive genome profiling techniques and new adaptive trials represent another more recent model, throughout the institution of Molecular Tumor Boards. Although the basic structure and framework are not yet homogeneous, they are nonetheless becoming more useful and widely adopted decision tools, and a Guidance document has been recently approved by the Italian National Agency for Health Services.

Models for integrating immunotherapy in healthcare are currently limited and not established. Existing literature predominantly draws upon experiences from individual medical centers rather than comprehensive integration models. In 2016, Champiat et al. presented a position paper with their 5‐year single‐center experience at Gustave Roussy. They focused on integration/multidisciplinary and defined five main phases of the process:

1. Prevent/basal evaluation: know the spectrum of toxicities—identify pre‐existing dysimmunity—inform patients and healthcare providers—identify risk factors such as elderly, pregnancy, and chronic infections;
2. Early detection: low‐threshold clinical suspicion, active monitoring schedule;
3. Proactive surveillance: close monitoring as oncologists are less familiar than with traditional toxicities;
4. Proper treatment: how to dose and how to taper corticosteroids—short‐ and long‐term sequelae—use of other immunosuppressors (e.g., TNF inhibitors in colitis)—when to resume or permanently discontinue ICIs (as to date there is no clear correlation between dose and efficacy or toxicity and indeed dose reduction is not recommended).
5. Monitoring: resolution kinetics of dysimmune toxicities, which can highly vary—the impact of immunosuppressants on disease response (as available data seem to show no negative impact on efficacy)—complications following immune‐suppressive such as opportunistic infections.^18^

This single‐center experience led the authors to establish a national pharmacovigilance registry (REISAMIC, Registre des Effets Indésirables Sévères des Anticorps Monoclonaux Immunomodulateurs en Cancérologie), committed to the collection of irAEs. In 2017, the Society of Immunotherapy of cancer (SITC) nominated a multidisciplinary Toxicity Management Working Group to develop recommendations for the standardization of the management of irAEs.^19^ The main goal of the workshop was to develop algorithms for the management of common and rare toxicities. Results represent the view of different experts in multiple fields, even if recommendations were driven in some cases by evidence from literature and in others by clinical experience and practice. Although evidence gaps were noticeable and a wide consensus was not reached on all issues, this working group led to useful statements on the main types of irAEs. In the absence of prospective clinical data and solid studies on predictive factors to date, the consensus recommendations provided a starting point that led to the development of SITC Clinical Practice Guidelines, based on the best available literature evidence, as well as clinical experience and expert opinion, where appropriate. In the following years, several guidelines including the American Society for Clinical Oncology (ASCO), the European Society for Medical Oncology (ESMO), and the National Comprehensive Cancer Network (NCCN) were developed. These are now regularly updated as clinical practice burdens and data from clinical trials grow. Only in the last 3 years, in parallel with emerging evidence and guidelines implementation and as the need for multidisciplinary became more evident, the number of studies on retrospective and single‐center multidisciplinary teams has grown. In 2019 Naidoo et al. aimed to determine whether a virtual immuno‐related toxicity (IR‐tox) team would be feasible to implement and be used in clinical practice. Patients treated at the Sidney Kimmel Comprehensive Cancer Centre at Johns Hopkins Hospital with ICIs and referred to the team from August 2017 to March 2018 were considered. IR‐tox team was found to be feasible and useful: 117 referrals coming from 102 patients were received in 8 months, all providers received recommendations within 24 h, 100% of providers used recommendations and in 74% of cases patient's management changed based on IR‐tox team recommendations.

Notably, this study provided an electronic referral template, even if both team interaction and patient management were face‐to‐face. These results confirm that such an electronic platform could be used by multiple specialists, reduce administrative burden and help spread expertise to limited‐access settings (from inpatient to outpatient). The authors concluded that the study could represent the basis for future studies designed to evaluate whether an IR‐tox team approach versus ad hoc specialist referral could influence patient outcomes, such as hospitalization rate, duration of corticosteroids intake, and discontinuation rate.^20^ Similarly, in 2018–2019, an immune toxicity working group was established at Dana‐Farber Brigham and Women's Hospital, with the subsequent launch of the Immune‐toxicity (ITOX) Service. In 2020, Abu‐Shawer et al. presented retrospective data from the one‐year experience of this inpatient service, characteristics, clinical patterns, and management confirming the feasibility of an Electronic Medical Record (EMR) to triage patients to a specialized team.^21^ Similarly, in 2017, the Massachusetts General Hospital implemented the Severe Immunotherapy Complications (SIC) Service, a multidisciplinary team for hospitalized patients with irAEs committed to a clinical‐translational effort to study and treat these novel toxicity patterns.^22^ Lately, in 2021, Zubiri et al. presented a study to evaluate the impact of interventions on patients' outcomes and healthcare utilization. A hospital database was used to identify patients who were hospitalized for severe irAEs before (April 2016–October 2017) and after (October 2017–October 2018) SIC Service initiation. The primary outcome was the readmission rate after hospitalization. Secondary endpoints included length of stay (LOS) for admissions, use of corticosteroids and non‐steroidal second‐line immunosuppressants, ICIs discontinuation, and inpatient mortality. In the pre‐SIC period, 127 of 1169 patients treated with ICIs were hospitalized for irAEs. Differently, in the post‐SIC period, 122 of 1159 were admitted. After SIC initiation, the readmission rate dropped (14.8% post‐SIC vs. 25.9% pre‐SIC; OR 0.46; 95% CI 0.22 to 0.95; *p* = 0.036) and readmission LOS was shortened (median 6 days post‐SIC vs. 7 days pre‐SIC; 95% CI −16.03 to −0.14; *p* = 0.046). No significant differences were detected in the use of corticosteroids and second‐line immunosuppressants, ICIs discontinuation and inpatient mortality rates. Reduction in readmission rate could be of particular relevance both for patients and hospitals as this is a commonly used measure for quality of care. Indeed, recently in the United States, the Hospital Readmission Reduction Program has begun penalizing hospitals with high 30‐day readmission rates. When the study was conducted, the feasibility of “irAEs‐toxicity” services had been already demonstrated but evidence about the impact on clinical outcomes was not yet reported. In this study, the authors evaluated the impact of switching from a traditional clinical practice model to an innovative clinical–translational research one. However, this study has several limitations. First, this is a single‐center experience, Second, this study was conducted in an academic medical center where the authors were able to recruit numerous specialists interested in irAEs management. Third, a general improvement in knowledge and experience in managing irAEs may have led to the observed differences in the pre‐SIC and post‐SIC groups. Despite these potential biases and the retrospective design limitation, to date, this is the first study reporting that a highly specialized care team could contribute to improving patients' outcomes. Moreover, the creation of a centralized blood and tissue bank could allow for further investigate of the molecular mechanisms underlying irAEs and identify predictive biomarkers.^22^

In summary, evidence available from a review of the literature so far shows retrospective, single‐center case series reports. Taking together all these reported experiences the design was essentially retrospective. The irAEs Teams described were heterogeneous among different experiences and different institutional protocols, and they were created in the context of single institution. Data so far reflects single‐patient tailored management with no standardization, and end‐point assessment was mainly focused on the inpatient setting (e.g., hospitalization rate, morbidity), with no “cost‐effectiveness” analyses presented yet.

## 3 PROPOSAL OF AN ITALIAN REGIONAL MODEL

The Oncology Management Fast Track (OMFT) is a two‐year postgraduate course on healthcare management in Oncology held by SDA Bocconi in Milan, involving young Oncologists from main Italian institutions.

At the end of the 2020–2022 course, held during the SARS‐CoV‐2 pandemic, we decided to design an inter‐institutional project to gather issues of hot debate and hot topics from both clinical Oncology and Healthcare management. We set the main topics, respectively, in the identification of models for the treatment of new toxicity profiles which are not yet clearly defined and require multi‐specialty integration, and in the search for novel strategies balancing hyper‐specialization and network integration in limited‐resources contexts, that could overcome the mere “in‐person” frame through the opportunities offered by accelerated telemedicine implementation.

The project is aimed at coordinating and integrating the practices of two different hospitals into a single collaborative model to manage the irAEs. Organizational trust frameworks between the two institutions are different but potentially complementary with possible synergisms: Polyclinic of Milan is a Higher Tertiary Referral Centre for the entire administrative region of Lombardy and has been designated as an Italian Scientific Research and Treatment Institute and Multi‐Specialty Hospital. Conversely, the ASST Bergamo Est Trust is a multi‐hospital structured trust formed by several net‐working General District Hospitals spreading out/stretched across the territory of the Bergamo District area, with its articulated structure on the territory of the Province of Bergamo (with a catchment area extended over 94 municipalities and to a total population of 387,000 inhabitants).

During the pilot phase between 2020 and 2021, reference specialists in the different disciplines dedicated to the management of immunotherapy toxicity were identified within the Polyclinic of Milan for each area of relevant expertise; each of these appointed HCPs was asked to record a talk and set up a slide presentation focused on their area of interest for irAEs management, subsequently included in an online intranet webinar. They issued Trust guidelines that merged and have been recently published within the Trust as the “Manual for the Management of Immunotherapy Toxicity.”^23^ To define the dimension of need underlying the project, first, we retrospectively revised all the clinical histories of patients who had received immunotherapy in the two Oncology Departments involved in the project for the period September 2019–September 2021. Audit of the initial series from the two separate departments allowed us to assess the feasibility and the clinical burden and relative administrative requirements for project implementation. Relative to the Milan Polyclinic, 37 patients out of 180 who received immunotherapy reported at least one toxicity. Three cases required hospitalization for severe toxicity (grades 3 and 4), with two deaths. All the remaining cases were managed on an outpatient basis. Differently, 68 patients were treated in the same period at ASST Bergamo Est, of whom 18 reported irAEs of any degree. Three cases required hospitalization via the Emergency Department (one for G3 diarrhea with dehydration and pre‐renal acute renal failure, another case for interstitial pneumonia, and the last case for autoimmune pericarditis); there was a single mortality reported in the series. These data are aligned with the above‐reported literature. Based on retrospective analysis of these pilot series and with limitations of small sample size groups, a caseload of 40 cases for Milan and 20 cases for Bergamo on an annual basis was predicted. The reference population is expected to consist of patients with all the major solid neoplasms of the adult of higher incidence and prevalence and for which immunotherapy is currently indicated in the first line or later lines, with particular reference to pulmonary, genitourinary, gastrointestinal, and ENT neoplasms.

The project intends to enable standardization of patient management, avoiding patient troublesome transfer across hospitals through the implementation of telemedicine models and specifically through teleconsulting. This would be performed by using an existing telematic platform for specialists' cooperation. The team structure is defined as a working network, the core team, consists of a medical oncologist, pneumologists, clinical immunologist/rheumatologist, endocrinologist, gastroenterologist, cardiologist, nephrologist, diabetologists, neurologists, and internal medicine specialists assisted by the liaison figure of a case manager. The team referrer is the medical oncologist, a pivotal figure as the first decision‐maker and manager of the patient's treatment pathway. The involvement of the laboratory and radio diagnostics as supporting clinical services is envisaged to ensure the rapid availability of the resources needed for a quick diagnostic process (blood tests, ultrasound, CT, and MRI).

**FIGURE 1:**
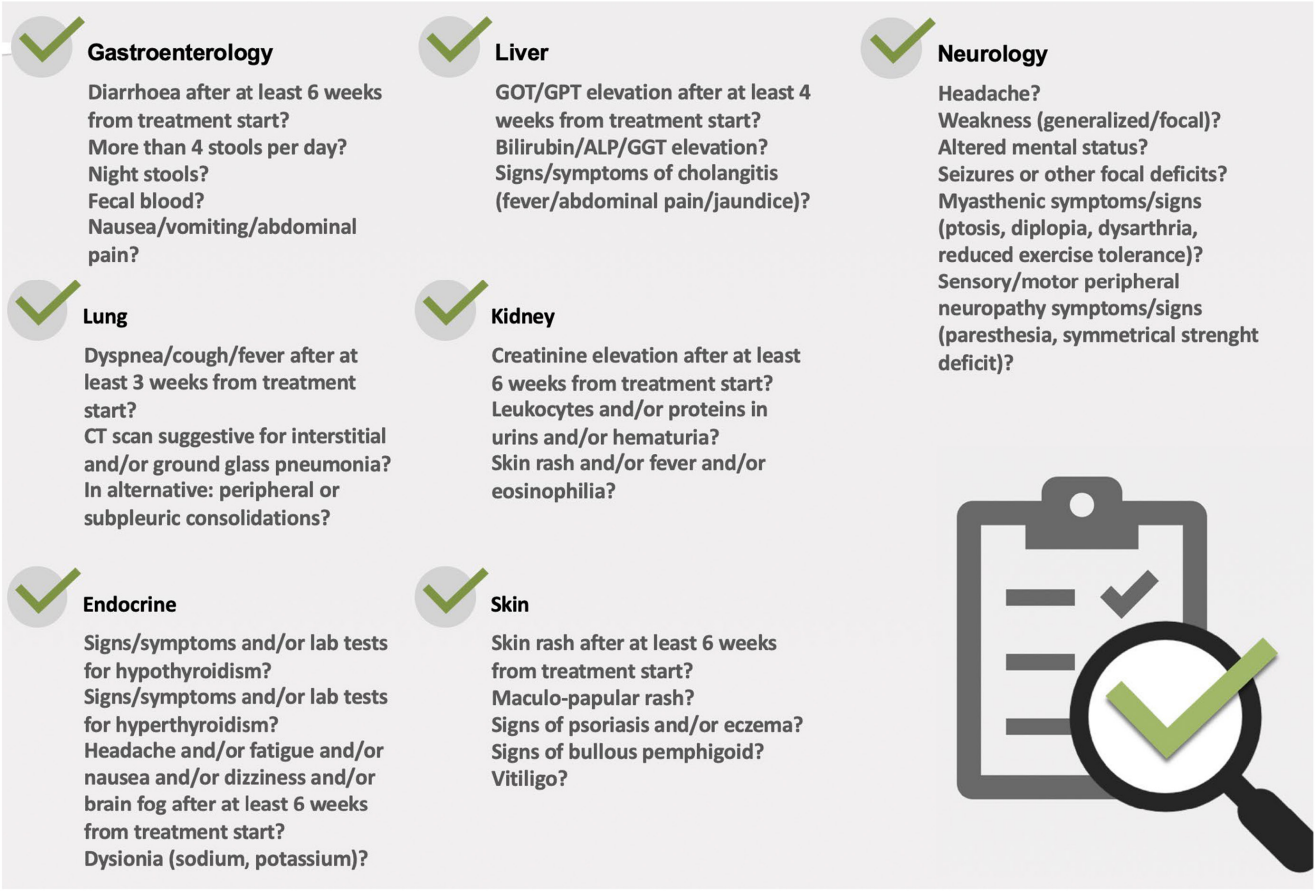
Operative checklist for suspected irAEs. ALP, alkaline phosphatase; CT scan, computed tomography scan; GGT, gamma‐glutamyl transferase; GOT/GPT, liver transaminases.

The model shape is a multi‐professional and multidisciplinary cooperative network with an adaptive and on‐demand configuration to deliver agile assistance according to clinical needs. The management pathway starts with the creation of a simplified form for the collection of the patient's demographic and clinical data (pathology stage indication and line of treatment—baseline assessment with any risk/comorbidity factors), filled in by the oncologist in each of the two centers. Second, the specialist has to deal with a simplified “checklist” (Figure 1), structured in two sections, for the patient (e.g., symptom severity, onset) and clinician (e.g., physical examination findings, laboratory data) to fill in respectively.

This report should be descriptive of any symptoms reported and their degree/intensity during the intervals between administrations. The leading oncologist first evaluates clinical conditions; at this stage, team intervention is triggered and teleconsultation is planned. Most irAEs can indeed be identified and graded based on laboratory findings and “patient‐reported” symptomatology (collected through the checklist), and thus do not require the physical presence of the patient. Moreover, through peer‐to‐peer communication and without the need to identify a predefined day and time, this referral pathway allows for a more immediate, streamlined, and “on‐demand” case referral between oncologists and other specialists; such a model could be effective particularly when time‐critical interventions are paramount for good outcome (as in emergency setting). The project will undergo a three‐staged service evaluation process. After the first phase structured as a pilot project for a general setting, outcomes analysis, data collection, and identification of emerging critical issues, it is planned to define appropriate indicators for a second phase of implementation. Upon the eventual positive outcome of the second phase and the resolution of any critical issues, then in the third phase, the proposal for the enlargement of the network with the extension to more centers in the context of the Regional Health Service through the involvement of the reference Institutions (AIOM Lombardy, CIPOMO) is envisaged.

**FIGURE 2:**
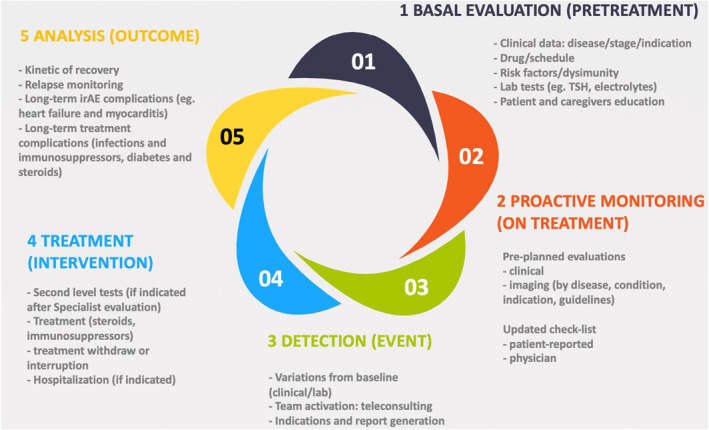
irAEs management flow‐chart. TSH, thyroid‐stimulating hormone.

In its first (pilot) phase, the project starts with the “baseline pre‐treatment assessment” of the patient. The simplified clinical data/data form completed by the clinician should report pathology data, indication, and line of treatment. Then, the drug schedule and spectrum of major irAEs are taken into account together with any risk factors and predisposing conditions. The baseline clinical/laboratory parameters (e.g., baseline TSH‐r, recommendation weak positive AIOM 2020 guidelines) should also be considered. A “proactive surveillance” is warranted during the entire treatment period. The referral oncologist should plan a prearranged clinical assessment at each access for treatment or scheduled diagnostics re‐evaluations as per guidelines. Moreover, the checklist should be updated at each access: completed by the patient for symptoms (patient‐reported) and by a clinician for any signs and laboratory data. In case of a possible diagnosis of irAEs, the possible deviation of clinical/laboratory parameters from baseline should be reported. Moreover, the team should activate teleconsultation on the identified platform to generate and share a report with indications for intervention. In case treatment is needed, any additional level II laboratory or instrumental investigations should be performed. Moreover, the team may decide on treatment interruption and/or steroids or immunosuppressant administration. If needed, possible hospitalization should be considered. In the end, a “clinical outcome analysis” should be performed. Among variables, resolution kinetics, possible recurrence, long‐term complications of the event (e.g., acute renal failure after high‐grade diarrhea, cardiac decompensation after myocarditis) and long‐term complications of immunosuppressive treatment (e.g., the incidence of infectious events, diabetes secondary to steroids, etc.) must be taken into consideration (Figure 2).

On the whole, the first phase has three macro‐objectives: first of all, it should carry out an improvement of clinical outcomes/care efficiency (reduction of the rate of definitive discontinuation of treatment). Second, work should determine a reduction of admissions to the ER for irAEs and, as a consequence, a reduction in the number of hospitalizations. The third goal is identified as the collection of a database on case histories and outcomes, for the identification of critical issues and the design of indicators for the second phase of implementation, as well as for possible subsequent publications. The expected outcomes of the initial phase are two‐fold. First, the establishment of an interagency and inter‐specialty working team is prioritized. Additionally, the first phase aims to introduce an innovative telemedicine model tailored to managing complications for oncology patients. This model aims to optimize care delivery and enhance clinical outcomes through the three stated macro‐objectives, facilitating the transfer of knowledge and skills and positively impacting the quality of care. Additionally, the project will enable the expansion of the two oncology units' series, creating a database for future observational studies and publications.

Expected outcomes of the second phase involve the analysis of the outcomes of the first phase with the identification of critical issues and proposals for adaptation. Moreover, in the second phase, key performance indicators (KPIs) should be identified. In Table 1, possible outcome measures are reported.

**TABLE 1:** Proposed key performance indicators.

Setting	Indicator	Threshold/target
Quality—care efficiency	The interval between symptoms onset and team activation	<7 days
	Admissions/administered cycles in 6 months	<5%
Quality— admission rate reduction	Admissions/year	<50% pre‐team period
Structure	Validation of inter‐institutional flow‐chart (involvement of strategic directions)	NA
Process	Rate of activation within 72 h	>80%
Outcome	Rate of all‐grade irAEs resolving to grade 1 or less within 30 days	>80%

Expected outcomes of the third phase include critically analyzing the outcomes and indicators from the second phase and proposing the expansion of the network to additional centers within the Regional Health Service, involving relevant institutions such as AIOM Lombardy and CIPOMO.

## 4 DISCUSSION AND CONCLUSIONS

Immunotherapy is one of the most important innovations in the oncology field. Nonetheless, its emerging widespread diffusion is not always associated with the appropriate level of expertise and specific resources needed for adverse event management. IrAEs represent a new spectrum of toxicities that requires significant changes both in clinical practice and in the institutional framework of single Oncology units toward a wider network integration, merging higher multidisciplinary skills. Constant and strict cooperation between specialists and oncologists is mandatory to recognize, report, and safely treat organ‐specific toxicities.^24^

Until now, the published experiences have been rare, diverse, and primarily retrospective studies from US centers. In response, we have developed a multi‐institutional model for clinical governance and service line implementation in managing irAEs, involving two hospitals in Lombardy, the largest region of the Italian National Health Service (Servizio Sanitario Nazionale, SSN). This project aims to provide an operational framework based on our experiences in Lombardy, with the expectation that this model could soon be adaptable to other contexts within the Italian SSN. The prospective collection of patient information and their care pathways enables the gathering of evidence in areas of oncology practice that current guidelines do not adequately cover. Accordingly, we present a three‐phase project designed to investigate the safety and feasibility of the operational strategy and to measure relevant outcomes. Starting from the results from the pilot phase leading to the identification of critical features, the second phase aims to identify KPIs as outcome measures for both clinical and health‐economics issues.

Due to the lack of validated outcome indicators in the available literature, we identified proposed indicators for quality, structure, process, and outcome through inter‐institutional consensus. Here, we suggest targets and parameters for potential improvements in both clinical and healthcare management settings (Table 1).

The strength of this project is a multi‐level approach considering both clinical governance and patient management as integrated levels of the same framework system for irAEs. First of all, we considered surrogate indicators dealing with quality of care in terms of efficiency and admission rate reduction, and indicators concerning both the structure and the process of activation of the model have been evaluated. As an example, concerning indicators of clinical outcome we hypothesize to consider the rate of all‐grade irAEs resolving to grade 1 or less within 30 days, thus referring to patient candidate to resume immunotherapy. A second distinctive feature of the project proposed here is that it represents an inter‐institutional design involving two hospitals, unlike experiences in literature; to our knowledge, this is the first project reporting a multi‐institutional experience for change of service in irAEs management. Review literature to date consists of case‐series and single‐center institutional practice from Western highly specialized Tertiary, almost invariably in the US Massachusetts General Hospital with the SIC Service, Sidney Kimmel Comprehensive Cancer Centre at Johns Hopkins Hospital, ITOX Service at the Dana‐Farber Brigham and Women's Hospital.^20^, ^21^, ^22^

Policlinic of Milan and ASST Bergamo Est Trust represent different levels of publicly funded healthcare service in Lombardy territory, whose hospital network is designed to work in a coordinated and synergic collaboration to achieve gold‐standard care outcomes and guarantee optimal resource‐allocation as per regulatory mission in the Italian SSN. Policlinic of Milan is one of the biggest National Tertiary Referral Centers in Italy and also a University Hospital that combines a higher level of expertise and specialization with medical students' education and clinical research with the University of Milan. The ASST Bergamo Est is a large District General Hospital that provides Secondary Care for one of the largest catchment areas in Bergamo Province, with peculiar geographical and logistic characteristics. It serves the Bergamo Valley District which consists of a wide rural area and numerous country‐side towns. Our two institutions are to be considered complementary and work in cooperative synergy as required by the Italian SSN mission. For Policlinic of Milan it is important to constantly feed this specialist knowledge through an ever‐increasing number of patients, for the ASST Bergamo Est the project offers a better model to manage irAEs within its catchment area. In this context, the inter‐institutional formula would enable first the evaluation of the transferability of experience, and second, the dissemination of skills that can impact on improving quality of care and clinical outcomes.

The project, in the third phase, aims to expand our networking model to more hospitals in the regional or even national system, involving the main institutional stakeholders. This is why the identified KPIs could be exportable to other institutions and a regional and/or national scale, representing in this way a unique feature.

A third peculiarity of the project is set on the telemedicine‐based formula: while previous experiences such as the SIC Service implied specialists' meeting in person, we took advantage of the compelling recent pandemic crisis that severely hit Lombardy in general and our hospitals in particular, and making the most of the compulsory telemedicine implementation we designed a faster, lean and flexible Multidisciplinary Team model based on teleconsultation. This would guarantee an “on‐demand” and agile response, overcoming the issues of geographical distance and specialists' scarcity.

Lastly, reported experiences such as the SIC Service were conceived for the management of irAEs in the inpatient setting, as in the US healthcare system the hospitalization and readmission rates are frequently used measures for quality of care. Conversely, although it may also be viable for inpatients, our model is built mainly on the outpatient setting, which in the Italian healthcare system represents the vast majority of patients treated in oncology units in general, and with immunotherapy in particular.

With its characteristics, the multidisciplinary management of irAEs based on telemedicine fits into the debate on the renewal of healthcare systems, the search for balance between hyper‐specialization and integration, and the push for change toward multi‐professional and multidisciplinary healthcare focusing on the rising use of telemedicine precipitated by the pandemic experience.

Considering the rapid pace of clinical innovation in oncology, professionals are tasked with both the responsibility and the opportunity to suggest new organizational strategies to effectively navigate the impact of these innovations. Given the limited research evidence in this dynamic landscape, these novel models have spurred comprehensive organizational research within Health Services.

## AUTHOR CONTRIBUTIONS

**Alessandro Iaculli:** Methodology (equal); writing – original draft (equal); writing – review and editing (equal). **Michele Ghidini:** Methodology (equal); writing – original draft (equal); writing – review and editing (equal). **Francesco Locati:** Methodology (equal); supervision (equal). **Laura Chiappa:** Supervision (equal); validation (equal). **Giuseppe Nastasi:** Data curation (equal); formal analysis (equal). **Gianpiero Fasola:** Resources (equal); supervision (equal); validation (equal). **Francesco Grossi:** Investigation (equal); supervision (equal); validation (equal). **Ornella Garrone:** Funding acquisition (equal); investigation (equal); methodology (equal). **Valeria D. Tozzi:** Methodology (equal); supervision (equal); validation (equal); writing – original draft (equal); writing – review and editing (equal).

## FUNDING INFORMATION

This study was supported by the Italian Ministry of Health (Ricerca Corrente 2024). The funders had no role in study design, data collection, analysis, and interpretation, or preparation of the manuscript and decision to publish.

## CONFLICT OF INTEREST STATEMENT

The authors declare no competing interests.

## ETHICS STATEMENT

With this paper, we propose a model of a future observational study. Please note that neither patients were enrolled nor clinical data regarding patients were collected.

## CONSENT

Not applicable.

## Data Availability

Data sharing not applicable to this article as no datasets were generated or analyzed.
